# The Importance of Using Physical Tridimensional Models for the Management and Planning of Extended Osseous Odontogenic Lesions

**DOI:** 10.3390/dj9110134

**Published:** 2021-11-15

**Authors:** Domenico Guerra, Marco Severino, Sara Caruso, Sofia Rastelli, Roberto Gatto

**Affiliations:** Department of Clinical Medicine, Public Health, Life and Environmental Sciences, University of L’Aquila, 67100 L’Aquila, Italy; marco.severino@student.univaq.it (M.S.); sara.caruso@student.univaq.it (S.C.); sofia.rastelli@student.univaq.it (S.R.); roberto.gatto@univaq.it (R.G.)

**Keywords:** oral pathology, odontogenic keratocyst, histology, enucleation, oral surgery, case report

## Abstract

(1) Surgical intervention becomes crucial in situations in which lack of action would cause a decrease in quality of life for the patient. As healthcare professionals, our next objective is to reduce patient fear perception. This work’s aim is to illustrate how physical tridimensional models can serve not only as confidence boosters for the patient, but also as a valid tool to aid both the clinician and the fostering of a patient–doctor relationship. (2) An example case managed using a stereolithographic model in the pre-surgical planning stage is presented in which surgical planning was carried out by analysis of radiographic investigations combined with a tridimensional resin model derived from the patient’s x-ray exam. (3) Successful enucleation, surgical debridement, and stable follow-up shows the effectiveness of the applied surgical protocol, confirming that planification using a physical representation of the tridimensional exam aids in the correct surgical management of said lesions. (4) The effectiveness o101f the surgical act itself as well as the follow-up showing ossification of the bony lesion and absence of relapse of a highly recurrent lesion confirms the effectiveness of the tools used for this surgical intervention.

## 1. Introduction

Advancing technology leads to the improvement of current methods and practices, where advancement is applicable. The expansion and growth of technology has allowed for the possibility of creating virtual models of situations that come very close to reality both in terms of their representation and fluidity. Aerospace trainees, as well as military forces, automobile companies, and videogame producers, utilize technology to give the user the feeling of experiencing reality. Healthcare professions should also take advantage of technological advancements. In 1927, doctor William J. Mayo stated that “there is no excuse today for the surgeon to learn on the patient”. According to the book *To Err Is Human* [1], 98,000 patients in the U.S. alone and as many as 1,200,000 patients worldwide die due to preventable reasons (data collected by Agency for Healthcare Research and Quality). This would place human error as the third leading cause of death in the United States [2]. The use of technology to aid us in our day-to-day lives is especially crucial when dealing with patient quality of life. Therefore, the old saying “see one, do one, teach one” is no longer true and must be replaced with the more ancient quote by Confucius: “I hear and I forget, I see and I remember, I do and I understand”. To “do”, simulations have to work in such a way as to provide the user with the most realistic experience possible. In the past, virtual simulations were the only tool available. During 1960s, the Resusci Anne doll was used to teach cardiopulmonary resuscitation (CPR) in a practical fashion. Tridimensional printing has come a long way since its inception in 1986. The aim was to bring simulation from the virtual world to the physical world and to then translate it into real situations. 

Stereolithography is an additive manufacturing technology by which a radiographic scan, acquired from such technologies as cone beam computed tomography (CBCT) or magnetic resonance imaging (MRI), can be converted into a physical model of the studied area [3]. These exams convert a physical object or person into digital information, which is saved as a digital imaging and communications in medicine (DICOM) file. These files are then processed and converted into standard tessellation language (STL) files, which can be analyzed by the 3D printer to produce an almost perfectly accurate tridimensional reproduction of the analyzed structure(s) [4]. By ejecting several layers of different resins onto a tray and curing them instantly, the machine can construct a model that matches every minute detail with more than one material at a time. For this reason, the produced models have similar densities to those of the areas analyzed, with different structures being represented by resins with different densities. For example, nerves or tumors are soft tissues and therefore much less dense than bones—and are represented as such in terms of the density of the resins used. All these characteristics give the feeling of operating on an actual patient, making this an indispensable tool for surgeons who want to better understand and plan a surgery. 

Such a tool becomes of great importance when planning and recreating a particular surgical procedure. Adapting their surgical technique based on the patient’s personal anatomy and characteristics allows the professional to reduce surgical times, while simultaneously optimizing possible intra-operatory complications [5]. Apart from educational purposes, these models can be used when resective surgeries have to be performed in highly anatomically sensitive areas, such as neurologic passage landmarks and maxillary sinuses. Preventive planning is performed on the virtual 3D model [6], while planning is then performed on the physical model before the actual intervention takes place on the patient. Surgeons’ opinions regarding the benefits of stereolithographic model use as a pre-surgical tool confirm the optimization of the diagnostic as well as surgical act by means of reducing operating room time, improving visualization of the patient’s unique structures, and increasing pre-surgical commitment after surgical rehearsal on 3D models [5].

Among the many possible odontogenic lesions that may affect the jaw, two large groups can be distinguished, as described by the latest classification by the World Health Organization (WHO) (2017′s 4th classification [7]), based on their etiology. These two groups distinguish between lesions of inflammatory and developmental origin. The three main lesion types within these classifications are radicular cysts, dentigerous (follicular) cysts, and keratocysts. Keratocysts are odontogenic lesions of developmental origin, originating either from epithelial rests that arise from dental lamina and its remnants that are not reabsorbed after dental formation is completed or from offshoots of the basal layer of the oral epithelium. These entrapped epithelial remnants then undergo a proliferation that alternates between inflammatory and dormant stages, which over time define the expansion of the lesion itself and its anatomic surroundings [8]. Smooth bordered unilocular, scalloped border unilocular, or multilocular with smooth/scalloped border are some of the ways in which a keratocyst can be radiographically described and diagnosed. Predilection is strong in the mandible with the potential for cortical perforation during expansion as well as posterior relapse in case of lesion enucleation. Keratocystic odontogenic tumors (odontogenic keratocysts) are the main concern when a keratocyst is suspected. Their content is a characteristic “cheesy” material that represents the parakeratin produced by the luminal epithelium [9]. Treatment involves marsupialization or complete enucleation because of the extremely high relapse potential due to the left cystic tissue and lesion’s ability to form daughter cysts that are easily missed and will later cause lesion reactivation.

## 2. Materials and Methods

**Figure 1:**
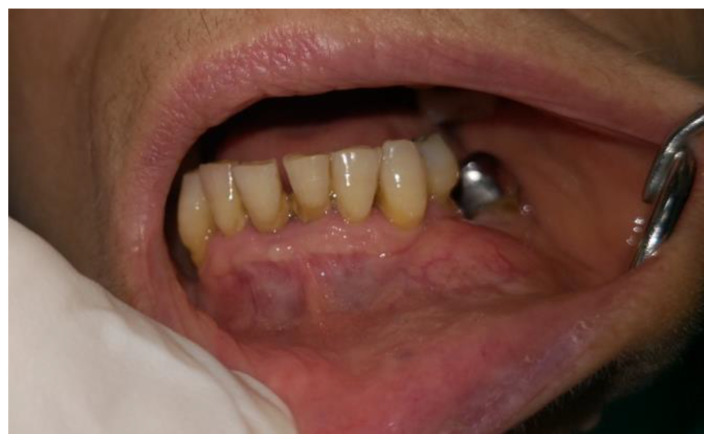
Intraoral osseous expansion.

A 45-year-old female patient presented at the Gubbio (Umbria, Italy) Public Hospital Dental Department with a swelling sensation in the lower left part of the chin. The patient said that she was not affected by any systemic pathologies and was not undergoing any pharmacological treatment. At the clinical oral inspection, a large bony expansion was observed located between element 3.3 and 3.4 but extending mesio-distally with respect to these elements (Figure 1). The absence of pain, either spontaneous or upon palpation, is noted.

**Figure 2:**
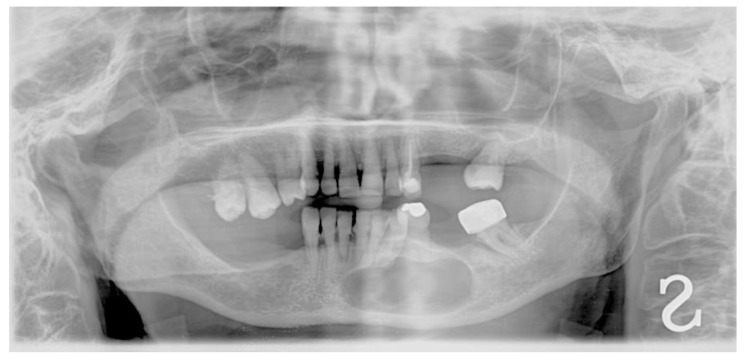
Initial OPG showing a wide radiolucent area in the mental area.

**Figure 3:**
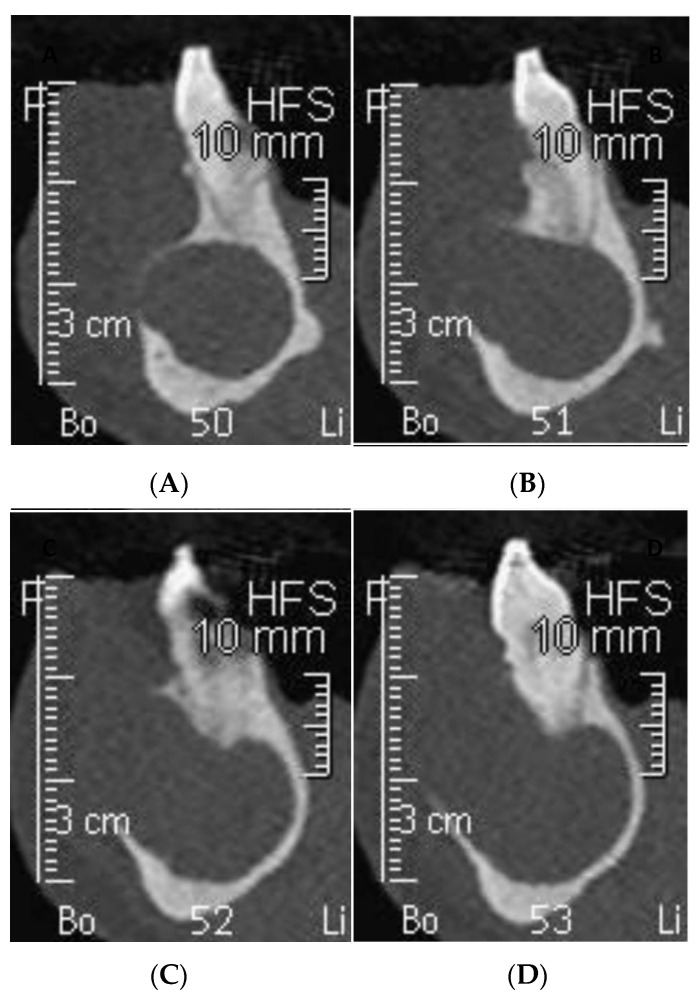
CBCT examination of sections between elements 3.3 and 3.4. (**A**) Distal aspect element 3.3 showing buccal osseous expansion. (**B**) Element 3.3, with evident buccal cortical bone erosion. (**C**) Increasing cortical opening due to cystic expansion. (**D**) Mesial of element 3.4 showing degree of buccal cortical bone elimination.

Orthopantomography (OPG) and cone beam computed tomography (CBCT) radiographic examinations were requested in order to evaluate the nature and extent of said lesion. One week later, the patient returned to the Dentistry Department to take the previously mentioned tests (Figure 2 and Figure 3), which evidenced a wide radiolucent area extending from the mesial of element 4.2 to the distal of the area corresponding to element 3.6, which is missing.

**Figure 4:**
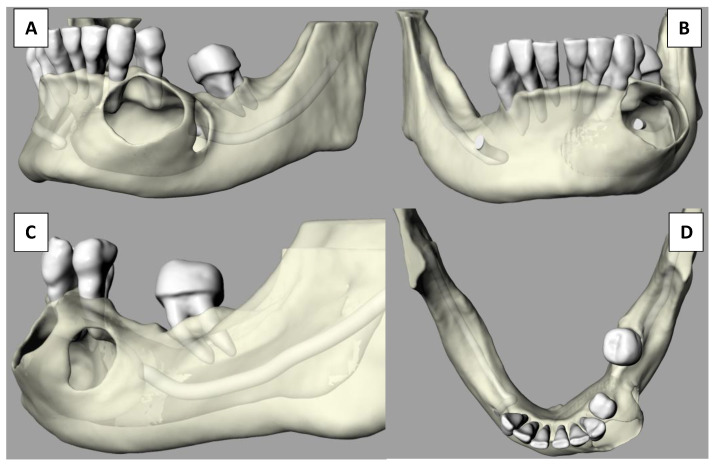
3D mandibular representation. (**A**) Left hemimandibular aspect. (**B**) Frontal aspect. (**C**) Lateral aspect. (**D**) Vertical aspect.

Differential diagnosis following radiographic examination was limited to ameloblastoma, odontogenic keratocyst, radicular cyst, and glandular odontogenic cysts due to their similar radiographic appearance. Due to the lesion’s possible malign nature, enucleation with histologic pathological confirmation was mandatory. The CBCT examination was digitally processed into stereolithographic files that were then imported into a digital printing program to be produced using a Multijet Connex350™ Multi Material Printer. The first step was the virtual reproduction of the lesion, which clearly shows buccal fenestrations in the regions of maximal expansion (Figure 4) as visualized in the CBCT.

**Figure 5:**
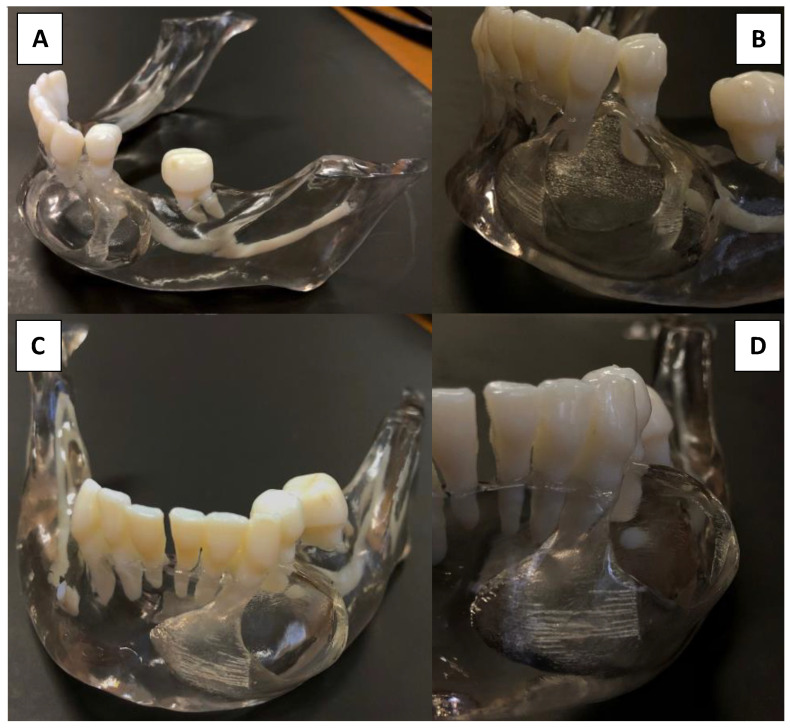
3D printed stereolithographic model. (**A**) Lateral model view. (**B**) Close image of lesion representation showing anterior and posterior cortical discontinuity. (**C**) Frontal view showing frontal cortical perforation. (**D**) Frontal view of the lesion showing mandibular nerve passage inside it.

Following tridimensional model acquisition, surgical planning was performed by visualizing perfectly anatomical landmarks and characteristics. The terminal branch of the inferior mandibular nerve (IAN) was completely engulfed inside the lesion, thus obliterating the mental foramen, the mental branch of the IAN, and 2 mm of the intraosseous IAN section. Sensitivity at the innervating regions was already altered before the surgery. The patient was presented with the 3D model of her mandible depicting the lesion and the planned surgical protocol was explained to her. Possible neurologic deficits were also explained, making sure the patient understood on the model the reason for the neurologic damage. Informed consent was obtained after the patient accepted the proposed surgical intervention that involved the extraction of five dental elements and a high probability of neurological deficits. The model was then utilized to plan surgical limits and to manage nerve transit inside the lesion (Figure 5).

**Figure 6:**
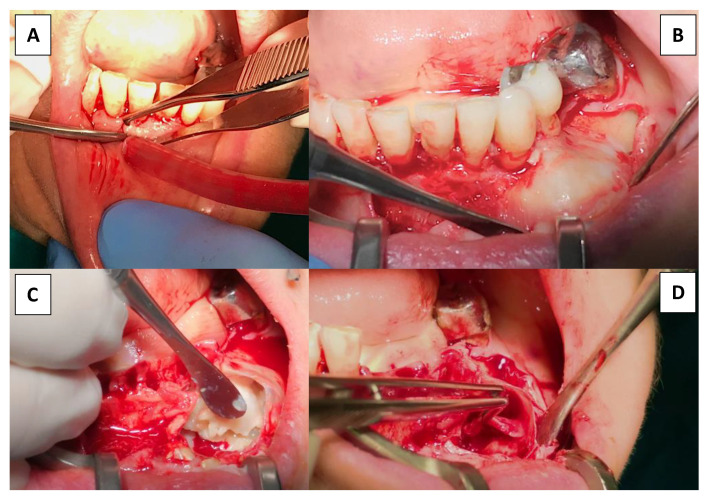
(**A**) Vestibular sulcular and releasing incision to access lesion. (**B**) Cortical bone expansion visualization following flap raise. (**C**) Cystic content before surgical emptying resembling cheesy appearance. (**D**) Mental nerve isolation during cystic detachment from its surrounding bony adhesions.

The patient was advised to begin antibiotic medication 24 h prior to the planned surgery, which would serve as prophylaxis due to the deep and extensive surgical field. Following local anesthetic administration, vestibular access was performed using intrasulcular incision of the existing teeth with two releasing incisions from the distal of element 4.1 to the distal of element 3.4 (Figure 6A).

The vestibular soft tissue flap was raised to expose the bone expansion and visualize the planned surgical field (Figure 6B). 

Evident vestibular cortical bone extrusion was observed, as foreseen on the model. Pathologic bone limits were isolated from the surrounding soft tissues. Although not directly related to dental causes, the lesion’s possible malign nature necessitated the complete removal of the pathologic tissue and everything that may cause lesion relapse. For this reason, five extractions, from element 3.4 to element 4.1, had to be performed. Enucleation of cystic content began from the mesial cortical perforation, from which the accumulation of keratin and parakeratin was removed, showing a characteristic cheesy appearance (Figure 6C). 

**Figure 7:**
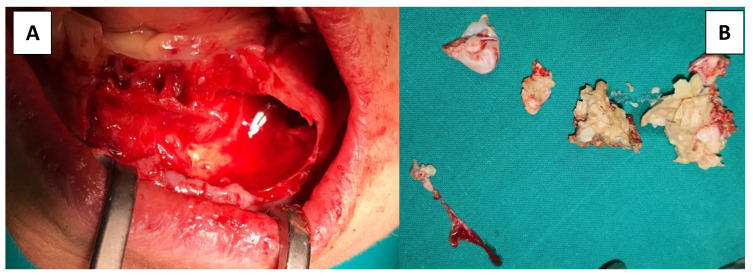
(**A**) Complete enucleation exposing the healthy bone perimeter. (**B**) Cystic capsule and its content later to be submitted for histologic evaluation.

Complete lesion content debridement was meticulously completed, including cystic capsule detachment from the surrounding cortical bone, ensuring complete elimination. Special care was taken during cystic debridement from its nervous relationship, and the nerve was found to be free inside the lesion. Pathologic tissue was carefully removed and the nerve delicately tractioned to remove lesion attachments along its trunk (Figure 6D). Bone contact was accomplished in every aspect surrounding the lesion borders to make sure of its complete enucleation. Clean bony walls are of great importance when performing such surgeries in order to prevent and reduce relapse potential (Figure 7A).

The cystic content (Figure 7B) was then immersed in formaldehyde solution in order to be submitted for histologic evaluation. The macroscopic characteristics from the histologic staining are crucial for the exclusion of lesions included in the differential diagnosis. This also aids the clinician in deciding on a specific post-surgical pharmacological administration and timetable, as well as scheduling follow-up control visits to evaluate recovery and evolution. 

Given the extended bone defect and the wide surgical field, sutures were applied to closely seal the created bone pocket from the crestal entry of infectious material. A loose suture was applied on the releasing incision mesial of element 4.2 in order to help surgical site drainage and avoid impaction of foreign material that could be the cause of profound osseous infection.

Sutures were to be removed in the second week post-operation to allow for primary soft tissue closure, protecting the open bone defect and allowing it to slowly heal during the six months following the operation. At the six-month follow-up, patient will be re-evaluated by performing clinical and radiologic examinations to assess hard tissue healing and maturation in light of a possible implant/prosthetic rehabilitation. Antibiotic medication for one week as well as systemic corticosteroid administration for three days was prescribed. Analgesic medication was also prescribed in case of post-operatory pain—to be taken only if needed.

## 3. Results

At the two-week post-operatory control session, for the suture removal appointment, the patient referred to a lack of sensation in the areas corresponding to the compromised innervating branch. Patient completely understood the reasons why lack of sensation had occurred and that she had preventively accepted visualizing from the model the degree of nerve damage. Since she felt a light tingle when stimulated, hopeful neurologic healing could be expected [10] but was not assured. At the 2-year follow-up visit, the patient reported complete healing as well as a complete restoration of neurologic function of the treated nerve section. The post-operatory compromised neurologic function was probably due to nerve damage or partial injury, judging by the full neurologic recovery within two years. 

**Figure 8:**
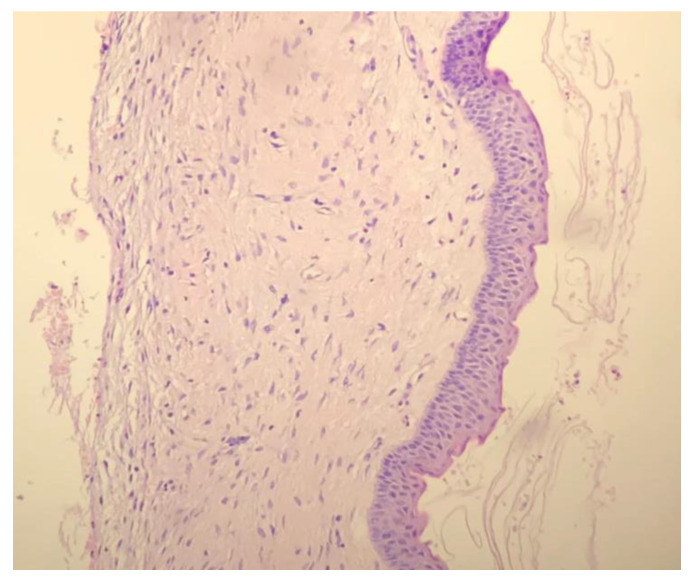
Histologic section of enucleated lesion confirming keratocystic formation (hematoxylin and eosin stain, original magnification 100.

From the histologic section (Figure 8), a stratified squamous epithelium cystic wall four to six layers in thickness [11] was observed, combined with the presence of cuboidal and columnar cellular infiltrates pointing the diagnosis towards a keratocystic lesion, which was confirmed by the presence of keratin. The absence of inflammatory infiltrate combined with keratin and parakeratin formation exclude all other possible conclusions from the differential diagnosis other than odonotogenic keratocyst.

**Figure 9:**
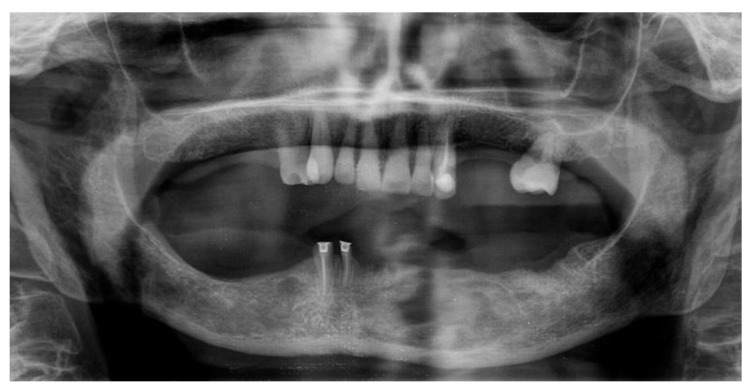
Two-year post operatory control OPG.

From the 2-year post-operatory control OPG (Figure 9), a wide radiolucent area corresponding to the cystic region is still observable. However, bone neoformation has certainly taken place as confirmed by the appreciated partial mineralization. The huge bone defect that was left behind will continue mineralizing for years [12] in an attempt to restore the initial bone condition. Full bone healing, however, will never be achieved and a “bone scar” will always be present and observable. Nevertheless, implantologic rehabilitation is an absolutely viable option for rehabilitation after careful case analysis using CBCT exams. Bone density and thickness is variable in this area and implant placement could aid in new bone formation [13] by reinforcing new bone bridges following implant osseointegration.

## 4. Discussion

Due to the multiple diagnostic spectra being narrowed down in the differential diagnosis phase, many of the considered lesions had a very important mutagenic potential. Of the many considered pathologies, odontogenic keratocyst was the most benign and the one with best prognostic factors. Thanks to the tridimensional imaging tools, important information was retrieved regarding the precise location, anatomical landmarks, and lesion density. Moreover, the compromised neurologic trunk was well depicted and therefore easily understood by the patient, who fully accepted the possibility of neurologic sensitive sequelae. Thanks to the modelling technology, therefore, the clinician was able to gain a better understanding of the surgery being planned, as well as help the patient visualize the intervention that was being proposed [14]. The importance of these tools and the aid they provide is extensively supported in the literature, which demonstrates that as this technology keeps progressing, more and more professionals start implementing it in their pre-surgical planning [15]. The value of this tool spans many different surgical fields, finding application from small surgical interventions to extensive neurosurgical planification, demonstrating the deep value these tools are acquiring for surgeons of varying degrees of expertise [16]. Oral surgeons are experiencing an improvement in the predictability of their clinical outcomes, a reduction in operating time, and the possibility of reducing morbidity in the patient; this is all evidence that 3D models are gaining more attention due to their technological and educational value [17]. Neurologic damage is a very common factor in many legal disputes, giving the use of 3D models to explain a sensible surgical intervention and the acquisition of informed consent another level of importance. The possibility of including 3D models in every anatomically limiting intervention could minimize legal disputes and further improve patient–clinician relationships and trust. Even though surgery was completely planned and reviewed on the model before the actual intervention took place, slight neurologic injury was inevitable considering the complete ablation of the mental foramen and therefore the absence of its terminal trunk. However, full recovery of the area after 2 years could not have been possible without visualizing how the nerve ran across and through the lesion with the use of the model, which allowed for the meticulous planning of its least traumatic enucleation. The limitations of 3D models, such as the inability to visualize soft tissues, can be made up for as the tissues can be examined by changing the contrast options on the preoperatory CBCT analysis. Unfortunately, no post-operative CBCT was taken since OPG offered enough information regarding site healing and since no implant-supported rehabilitation was planned; therefore, when following the ALARA guidelines [18], CBCT was of marginal importance.

## 5. Conclusions

In conclusion, successful surgical treatment was given by the best predicted outcome, allowing for minimal expected complications. Diagnosis and planning are key steps to follow in order to provide maximal advantages and limit the disadvantages—variables present in every complex surgical act. The ability for the surgeon to work on a familiar anatomical field by having examined it on a tridimensional representation gives a higher chance of limiting the disadvantages caused by unexpected intra-operatory complications. Moreover, patient understanding and full acceptance of what would otherwise be an abstract treatment explanation further aids in lowering the surgeon’s intra-operatory stress in light of possible and plausible legal disputes. Therefore, tridimensional models, as presurgical planning tools, directly and indirectly affect patient and surgeon behavior towards the given planned intervention.
